# Dendritic Cell Plasticity in Tumor-Conditioned Skin: CD14^+^ Cells at the Cross-Roads of Immune Activation and Suppression

**DOI:** 10.3389/fimmu.2013.00403

**Published:** 2013-11-25

**Authors:** Rieneke van de Ven, Jelle J. Lindenberg, Dinja Oosterhoff, Tanja D. de Gruijl

**Affiliations:** ^1^Department of Medical Oncology, VU University Medical Center, Cancer Center Amsterdam, Amsterdam, Netherlands; ^2^Laboratory of Molecular and Tumor Immunology, Robert W. Franz Cancer Research Center at the Earle A. Chiles Research Institute, Providence Cancer Center, Portland, OR, USA

**Keywords:** dendritic cells, human DC subsets, skin, macrophages, cancer, immune suppression

## Abstract

Tumors abuse myeloid plasticity to re-direct dendritic cell (DC) differentiation from T cell stimulatory subsets to immune-suppressive subsets that can interfere with anti-tumor immunity. Lined by a dense network of easily accessible DC the skin is a preferred site for the delivery of DC-targeted vaccines. Various groups have recently been focusing on functional aspects of DC subsets in the skin and how these may be affected by tumor-derived suppressive factors. IL-6, Prostaglandin-E2, and IL-10 were identified as factors in cultures of primary human tumors responsible for the inhibited development and activation of skin DC as well as monocyte-derived DC. IL-10 was found to be uniquely able to convert fully developed DC to immature macrophage-like cells with functional M2 characteristics in a physiologically highly relevant skin explant model in which the phenotypic and functional traits of “crawl-out” DC were studied. Mostly from mouse studies, the JAK2/STAT3 signaling pathway has emerged as a “master switch” of tumor-induced immune suppression. Our lab has additionally identified p38-MAPK as an important signaling element in human DC suppression, and recently validated it as such in *ex vivo* cultures of single-cell suspensions from melanoma metastases. Through the identification of molecular mechanisms and signaling events that drive myeloid immune suppression in human tumors, more effective DC-targeted cancer vaccines may be designed.

## Dendritic Cell Subsets and Their Plasticity in Human Skin: Impact on Cancer Vaccination

Skin is the largest human organ and its direct contact with the outside environment requires tightly regulated surveillance mechanisms to keep potentially harmful intruders at bay. For this purpose, human skin is densely populated with patrolling myeloid cells, such as Langerhans cells (LC) in the epidermal outer layer and various dermal dendritic cell (DDC) subsets and macrophages in the dermal layer (1, 2). It has been elegantly shown that different profiles of pattern recognition receptors present on the various myeloid subsets lining the skin makes them exquisitely specific in the recognition, uptake and either direct elimination of pathogenic microbes, or in presentation of pathogen-associated antigens for subsequent activation of the adaptive immune system (3–5). Interaction of a pathogen with pathogen-recognition receptors on dendritic cell (DC) induces activation of down-stream signaling pathways that result in their enhanced ability to process and present pathogenic antigens and in their migration to the draining lymph nodes, accompanied by phenotypic and morphological maturation, and priming of antigen-specific T or B lymphocytes (6). Whereas initially studies concerning DC subsets in human skin mostly involved the most predominant subsets, i.e., CD1a^hi^Langerin^+^ LC, CD1a^+^ DDC, and CD14^+^CD1a^−^DDC (7–9), the characterization of new surface markers and deeper phenotypic and functional analyses now show that further distinctions can be made (10–13).

**Figure 1:**
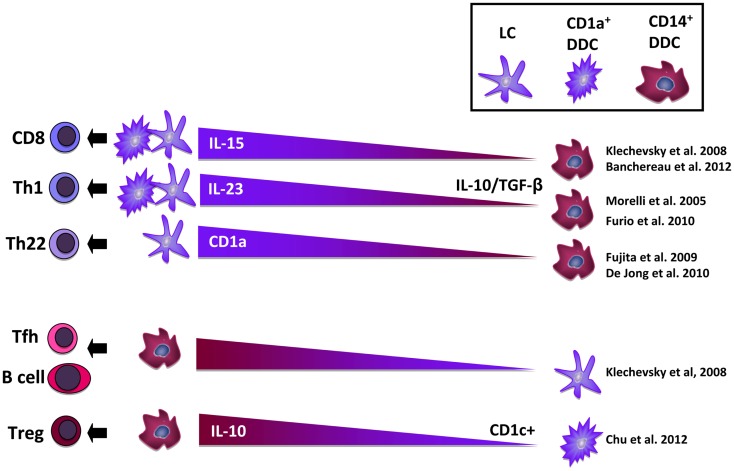
**Overview of the reported T cell differentiation induction abilities of mature Langerhans cells (LC) and CD1a^+^ dermal dendritic cells (DDC) vs. immature CD14^+^ DDC**. Abbreviations: DDC, dermal dendritic cell; IL, interleukin; LC, Langerhans cell; Th, T helper cell; Tfh, T follicular helper cell; Treg, T regulatory cell; TGF-β, transforming growth factor – β.

From our own work and that of others, it has become clear that beside epidermal LC and dermal macrophages at least five migratory DDC subsets can be distinguished (13, 14), i.e., CD1a^+^CD14^−^ DDC, CD1a^+^CD14^+^ DDC, CD1a^−^CD14^+^ DDC, and two double-negative subsets. An important issue that as yet remains unresolved is whether all these DC populations represent genuine subsets, or whether they are part of the same DC subset in various states of activation or differentiation. A growing number of studies now point to the existence of an inter-related population of cutaneous DC and macrophages in flux, trans-differentiating into each other as directed by environmental cues (8, 15, 16). This has direct consequences for the type of immune responses that will ensue, as different migratory DC sub-populations have now been directly linked to the induction of different types of immunity (13, 14) and have different capacities to cross-present antigens for the activation of cytotoxic CD8 T cells, a process crucial for the induction of anti-tumor immunity. Roughly, CD1a^+^ mature LC and DDC subsets have been linked to type-1 T cell mediated immunity, whereas CD14^+^ immature DDC subsets have been linked to the induction of humoral immunity and expansion of regulatory T cells (Treg) (11, 12); see Figure 1 for a schematic overview. Recent evidence suggests that tumors like melanoma abuse the balance between these subsets to effectively escape immune recognition (13, 17). In order for DC-targeted vaccines delivered through the skin to be effective, tumor-induced immune suppression should be overcome and T cell-stimulatory DC subsets selectively targeted. Here, we discuss mechanisms of tumor-imposed DC suppression in the skin microenvironment and how these may be counter-acted in aid of DC-based immunotherapy.

## LC and CD1a^+^ DDC: T Cell Activation

Klechevsky et al. first described a functional dichotomy between human LC and CD14^+^ DDC with the former preferentially activating CD8^+^ T cells and the latter B cells (9). In recent publications primary human LC have been shown to be superior inducers of Th22 cells (including conventional variant αβ-T cells restricted through CD1a) (18, 19). IL-22 has an important barrier function in homeostasis and safeguards the integrity of epithelial layers, but is also involved in pathological skin conditions like psoriasis. Furio et al. reported a superior ability of migratory LC over DDC to induce either Th1 or Th2 responses (20). Of note, DDC in this report consisted of CD1a^−^CD14^−^ double-negative DDC with a potentially lower capacity for T cell activation than CD1a^+^ DDC. Mathers and co-workers showed that while LC were superior Th17 inducers, human CD14^−^ DDC had the ability to skew Th cells to either a Th1, Th2, or Th17 profile, depending on their environmental conditioning, number, and activation state (21). To further delineate T cell-stimulatory properties of freshly isolated human LC vs. CD1a^+^ DDC, we undertook a genome-wide transcriptional profiling analysis which revealed CD1a^+^ DDC to express a far wider range of adhesion and co-stimulatory molecules, chemokines, and cytokines (and at higher levels), pointing to a putatively superior migratory and T cell stimulatory ability over LC in steady state conditions (22). Using a human cell line model of LC and CD1a^+^ DDC differentiation, we confirmed these data and showed DDC to be superior activators of cytotoxic CD8^+^ T cells. Importantly, this was validated in the same study by a comparative assessment of the *ex vivo* ability of human skin-emigrated LC vs. DDC subsets to prime HLA-A2-matched CD8^+^ T cells against an epitope derived from the MART-1 melanoma antigen (23). While LC and CD1a^+^ DDC were equally effective in priming allogeneic Th cells, DDC primed significantly higher rates of MART-1 recognizing CD8^+^ T cells at a higher functional avidity. Of note, Banchereau et al. have recently linked the superior effector CD8^+^ T cell priming capacity of LC and CD1a^+^ DDC to their release of IL-15 into the immunological synapse (12).

## CD14^+^ DDC: T Cell Tolerization

CD14^+^ migratory DDC are discernable from dermis-resident CD14^+^ dermal macrophages through their surface expression of CD1b and CD1c (24). In a comparative analysis with CD14^−^ DDC, CD14^+^ DDC were shown to be poor inducers of allogeneic T cells and to require high DC:T cell ratios for Th1 induction (25). This relative inability of CD14^+^ DDC to induce Th1 cells was related to their release of IL-10 and TGFβ1. We and others have found CD14^+^ DC to carry low levels of co-stimulatory molecules, to display a poor T cell priming capacity, and to be characterized by the expression of CD141/BDCA3 (Thrombomodulin), a marker that has been linked to a human DC subset with cross-priming ability (11, 13, 26). These CD14^+^BDCA3^+^ migratory DDC in a report by Chu et al. were shown to constitutively release IL-10 and to induce T cell hyporesponsiveness and Tregs (11). Moreover, they were able to cross-present self-antigens and inhibit skin inflammation in an *in vivo* transplantation model. These data point to an important role for this subset in T cell homeostasis. Banchereau et al. have pin-pointed the inability of CD14^+^ DDC to prime effector CD8^+^ T cells to their release of IL-10 and TGFβ (12) and the expression of Ig-like transcript 4 (ILT4) and ILT2 (27).

## Tumors Abuse DC Plasticity to Undermine Immunity: A Central Role for CD14^+^ DC

**Figure 2:**
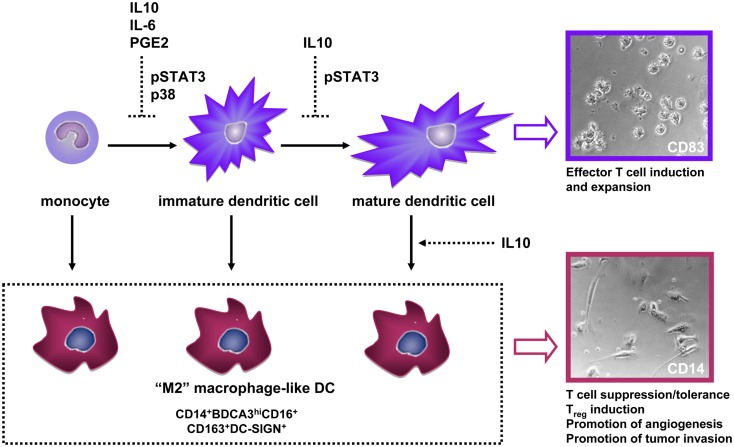
**Interference by tumor-associated soluble factors with normal dendritic cell (DC) development through indicated underlying signaling pathways, leads to (trans-)differentiation of CD14^+^ M2-macrophage-like cells with immune-suppressive and tumor growth- and invasion-promoting properties**. Photographic inserts illustrate the DC and adherent macrophage-like morphology of human skin-emigrated CD83^+^ and CD14^+^ DC, respectively (magnification 400×). Abbreviations: IL, interleukin; PGE2, prostaglandin-E2.

A large number of studies attest to the remarkable plasticity of the myeloid lineage; tumors abuse this phenotypic plasticity to re-direct myeloid differentiation toward the development of immune-suppressive subsets that effectively interfere with anti-tumor immunity (28). Consequently, tumors are often characterized by an infiltrate of immature macrophage-like cells and a lack of infiltrating DCs, which is generally a poor prognostic sign (28). We and others have shown that DC differentiation from monocytes can be blocked by tumor-derived soluble factors (most notably IL-10, IL-6, or PGE2) resulting in the development of CD14^+^ macrophage-like cells with poor T cell stimulatory abilities (so-called M2-type macrophages) and with T cell suppressive activity (Figure 2) (29–32). Beside monocytes, fully differentiated DC can be recruited to the tumor microenvironment, where they may lose their characteristic CD1a expression through the suppressive action of IL-10, as shown for melanoma metastases (33). A growing number of studies indicates the unique ability of tumor-associated IL-10 to convert even fully differentiated DC to CD14^+^ suppressive macrophage-like cells (8, 15, 16, 34, 35). IL-10 is generally expressed at high levels in the microenvironment of metastatic melanoma and can either be directly derived from tumor cells or from infiltrating immune cells. Among a panel of tumor-associated suppressive factors, we found IL-10 uniquely able to convert DCs to immature macrophage-like cells in two human model systems: (1) a physiologically highly relevant skin explant model in which we studied the phenotypic and functional traits of “crawl-out” myeloid cells (13) and (2) an *in vitro* model of tumor-conditioned DC maturation in which we functionally assessed CD14^−^ and CD14^+^ DC that had developed from monocyte-derived DC (MoDC) during IL-10-exposed maturation (17). In all above mentioned cases the tumor-induced M2-like cells shared some striking traits: an immature CD14^+^BDCA3^+^DC-SIGN^+^CD16^+^ phenotype and macrophage-like morphology (Figure 2), a disturbed balance in the release of immunosuppressive IL-10 (high) vs. immunostimulatory IL-12p70 (low), high expression levels of the T cell-inhibitory molecule B7-H1/PDL-1, and lower priming efficiency of allogeneic Th cells and of CD8^+^ (killer) T cells, the latter specifically recognizing the melanoma antigen MART-1, but binding epitope/MHC complexes with low avidity (13, 17, 32, 35).

In studies assessing CD1a and CD14 expression on DC from human skin explants, we showed the intracutaneous cytokine balance to be important for the subset composition of migrated DC (8, 13). Indeed, we have found compelling evidence that LC and CD1a^+^ DDC can actually trans-differentiate during and after migration from human skin explants to a CD14^+^ macrophage-like state in an IL-10-dependent fashion. Dermal conditioning by IL-10 or by topical application of irritants resulted in a shift among migrated DC from a mature CD83^+^CD1a^+^ state to an immature CD83^−^CD14^+^ macrophage-like state, passing through a CD1a^+^CD14^+^ intermediate stage (8, 15). Based on the fact that these CD14^+^ cells also expressed CD1c they were classified as DC rather than macrophages. Moreover, topical application of irritants to epidermal sheets showed that trans-differentiation from LC to macrophage-like DC depended on the presence of dermal fibroblasts and could be blocked by IL-10 neutralizing antibodies (15). A similar observation has been described in mice, where the presence of a subcutaneous tumor resulted in a DC-to-macrophage shift, with macrophage-like cells producing immune-suppressive factors such as IL-10, iNOS, and Arginase (16). Importantly, this trans-differentiation among DC that had migrated from human skin was preventable by co-injection of the DC-activating cytokines GM-CSF and/or IL-4 prior to skin explant culture (8).

Consistent with their expression of the M2-macrophage marker CD163, IL-10-converted CD14^+^ cells induced IL-10 and FoxP3 mRNA expression in allogeneic Th cells as well as a Th2-like cytokine profile and Treg expansion (13). Consistent with these tolerogenic qualities, IL-10-induced CD14^+^ macrophage-like MoDCs expressed high levels of immune suppression-related transcripts such as Indoleamine 2,3-dioxygenase (IDO), IL-4Rα, IL-6R, TGFβ1, HIF1α, and VEGFA (17). Activation of a HIF1α transcriptional signature has been reported in tumor-associated macrophages, even under normoxic conditions (36). This is in line with the transcriptional and cytokine release profiles we observed for CD14^+^ IL-10-conditioned DC, which revealed coordinated expression of HIF1α, TGFβ, VEGFA, MMP3, MMP9, IL-8, and TNFα, all of which can contribute to such tumor-promoting processes as endothelial cell migration and proliferation and tumor growth and invasion (28). In conclusion, tumor-related suppressive factors can divert DC during differentiation and even during and after maturation toward a macrophage-like state with immune-suppressive and pro-angiogenic and pro-tumor invasive properties (Figure 2).

Interestingly, in DC migrating from human skin, BDCA3 and DC-SIGN expression levels showed a very significant inverse correlation with CD83 maturation marker expression, indicating the utility of these markers for the identification of immature DC. Indeed, they marked CD14^+^ skin-emigrated DC as the least mature population with poor co-stimulatory properties (13). In keeping with these observations, DC that had migrated from skin explants taken from breast cancer mastectomy specimens, predominantly consisted of the CD14^+^DC subset with a macrophage-like morphology (13). Normalized distribution (i.e., more mature and less immature DC subsets) was observed for explants taken from patients that had received neoadjuvant chemotherapy: a clear indication that prevailing migration of the immature CD14^+^ subset was tumor-related.

From our observations we conclude that combined expression of CD14, BDCA3, DC-SIGN, CD16, and CD163 provides a phenotypic profile useful for the identification of M2-macrophage-like subsets with immune-suppressive and tumor-promoting characteristics that arise during tumor-conditioned differentiation or maturation of human DCs. We and others have found evidence of phenotypically similar subsets in breast, colon, head and neck, renal cell, and melanoma tumors (17, 37–39). Indeed, in single-cell suspensions derived from a panel of six metastatic melanoma tumors, we observed by multicolor flow cytometry analysis, that CD14^+^ cells, co-expressing both DC-SIGN and BDCA3 and detectable in a range of 1–38%, significantly outnumbered CD1a^+^ DC, which were virtually absent (ranging from 0.05 to 0.1%) (17). BDCA3 expression has recently been reported on skin-derived CD14^+^ DC that induced inflammation-attenuating Tregs (11). Combined with its association with cross-presenting DC subsets (10), this is highly suggestive of cross-tolerizing ability for BDCA3^+^DC. As yet, the functional significance of BDCA3/CD141 in either cross-presentation or immune suppression remains largely unclear, but some clues are emerging. Its Lectin-like domain can down-regulate NF-κB and mitogen-activated protein kinase (MAPK) pathways and might thus interfere with DC maturation and drive IL-10 release and Th2 skewing (40, 41). In keeping with this notion, BDCA3^+^ blood DC promote Th2 skewing (42) and *in vitro* generated or skin-derived CD14^+^BDCA3^+^ DC release elevated levels of IL-10 (11, 34). In addition, DC-SIGN can negatively impact DC activation resulting in prolonged and increased IL-10 transcription (43). Both DC-SIGN and BDCA3 may thus contribute to the immune-suppressive activity of tumor-modulated CD14^+^ cells.

Recently, the role of non-coding RNAs or microRNAs (miRNAs) in myeloid cell plasticity and functionality has also been studied. In mice, tumor-associated miRNAs were found to modulate the survival and longevity of DC (44), miR-223 was described to negatively regulate and miR-150 to positively regulate the cross-presenting abilities of LC (45, 46), the TGF-β associated miR-27a was reported to inhibit DC-mediated differentiation of Th1 and Th17 cells (47) and in an allergy setting miR-23b was shown to induce tolerogenic DC through inhibition of the Notch1/NF-κB pathway (48). In man, this field of research remains largely unexplored, though miR-155 was shown to regulate the M1/M2-macrophage balance by targeting the IL13-Receptor α1, thereby reducing M2 polarization (49).

## Signal Transduction Pathways Acting as Master Switches of Tumor-Induced DC Suppression: Targets for Therapeutic Intervention

Tumor-derived suppressive factors bind various receptors on myeloid cells but down-stream signals may converge in shared pathways. Mostly from mouse studies, the JAK2/STAT3 signaling pathway has emerged as a “master switch” of tumor-induced immune suppression (50). We have additionally identified p38-MAPK as an important signaling pathway in human DC suppression, and validated it as such in *in vitro* DC cultures and in *ex vivo* cultures of single-cell suspensions from melanoma metastases (32). From a panel of tumor-associated suppressive factors (including PGE2), we found only IL-6 and IL-10 to induce STAT3 phosphorylation during human MoDC development. As we had previously identified prostaglandins as the main culprit of suppressed DC differentiation by supernatants from single-cell suspensions of metastatic melanoma tumors (29) it was not surprising that STAT3 inhibition alone could not prevent this suppression; for this, combined JAK2/STAT3 and p38-MAPK inhibition was required. Importantly, combined interference in the STAT3 and p38 pathways completely prevented inhibition of DC differentiation by all tested tumor supernatants (*n* = 18, derived from both primary tumors and tumor cell lines, together encompassing eight different histological origins) and led to superior DC functionality, evidenced by increased allogeneic T cell reactivity with elevated IL-12p70/IL-10 ratios and Th1 skewing (32). Most importantly, combined STAT3 and p38 inhibition supported a shift from CD14^+^ monocyte-like cells to CD1a^+^ DC in metastatic melanoma single-cell suspensions, indicating a potential for improved DC differentiation in the tumor microenvironment (32). Of note, siRNA-mediated knockdown of STAT3 only, did effectively prevent the generation of CD14^+^ cells during IL-10-modulated MoDC maturation induction (17).

Altogether, these data point to different tumor-associated factors (i.e., IL-10, IL-6, PGE2) exerting their suppressive effects at various stages of myeloid DC development through converging and communicating signaling elements encompassing the JAK2/STAT3 and p38-MAPK pathways (Figure 2). To specifically address melanoma-induced myeloid suppression it is important to further dissect the JAK2/STAT3 and p38-MAPK pathways and possible cross-talk between them in melanoma-associated myeloid subsets in order to identify specifically acting and clinically relevant therapeutic targets. The advent of small-molecule kinase inhibitors and RNAi-based therapeutics now enables targeting not only of tumors, but also of their stroma, and should facilitate re-programing of tumor-associated myeloid cells, as well as tumor-modulated DC subsets in the skin, in support of anti-tumor immunity.

## Therapeutic Activation and Targeting of DC in the Skin and Its Lymph Catchment Area

Beyond the local suppressive environment at the site of the tumor, the immune-suppressive effects of the tumor stretch to draining lymph nodes where anti-tumor T cell responses should be primed. Sentinel lymph nodes (SLN) are the first-line tumor-draining lymph nodes and as such bear the brunt of melanoma-induced immune suppression (51). We have identified and characterized four conventional DC subsets in melanoma SLN, two of which were positively identified as skin-derived CD1a^+^LC and DDC, and the remaining two (CD1a^−^CD14^−^ and CD1a^−^CD14^+^) as LN-resident subsets with varying levels of BDCA3 and DC-SIGN expression (52). Deeper invasion of the primary melanoma in SLN tumor negative patients was related to a reduced activation state of skin-derived DC subsets in the SLN (53, 54). Also, lower frequencies of the skin-derived subsets were found in tumor positive SLN as well as a reduced activation state of LN-resident DC subsets (our own unpublished data). These findings indicate a local suppressive effect of the primary tumor on the activation state of skin-derived DC which then migrate to the SLN and lymph node metastasis-related suppression of SLN-resident DC subsets, and are in keeping with tumor-induced conditioning of the microenvironment (skin or SLN, respectively). Moreover, they suggest that primary melanoma-mediated suppression of activation and migration of skin DC enables local metastasis.

In two Phase II clinical trials we have demonstrated that localized intradermal administration of DC-stimulatory agents such as GM-CSF and CpG oligodeoxynucleotides (ODN), i.e., TLR9 ligands, led to increased activation of DC subsets in SLN of melanoma patients and tipped the local cytokine balance in favor of cytotoxic T cell mediated anti-tumor immunity (55–58). Although in man CpG ODN don’t directly bind to conventional DC, we nevertheless observed maturation induction of conventional DC subsets, most likely through CpG-induced cytokine release by plasmacytoid DC (57). In our human skin explant model we have similarly tested the effects of intradermal delivery of a panel of TLR-ligands on migratory DC and found a unique ability of the TLR2 and 3 agonists peptidoglycan (PGN) and polyriboinosinic-polyribocytidylic acid (Poly I:C) to enhance the T cell-priming ability of skin-emigrated DC, which, in the case of PGN, was accompanied by Th1 polarization (59). Surprisingly only small effects of the tested TLR-ligands on phenotypic DC activation were observed. This may have been due to induced IL-10 release, which might have been counter-acted by simultaneous signaling modulation (60, 61). Indeed, evidence for the therapeutic efficacy of combined STAT3 inhibition and CpG ODN was previously provided by Kortylewski and colleagues, showing superior immune stimulatory effects of CpG by eliminating collateral STAT3-mediated suppressive effects (62, 63).

In conclusion, JAK2/STAT3 and/or p38-MAPK signaling interference, combined with local immune potentiation, may counterbalance tumor-imposed suppression of skin DC subsets, minimizing the induction and trans-differentiation of migratory CD14^+^ M2-like DC with T cell suppressive characteristics, and thus set the stage for effective tumor vaccination through DC-targeted approaches.

## Author Contributions

Rieneke van de Ven and Tanja D. de Gruijl conceptualized and wrote the manuscript, Jelle J. Lindenberg and Dinja Oosterhoff co-wrote the manuscript.

## Conflict of Interest Statement

The authors declare that the research was conducted in the absence of any commercial or financial relationships that could be construed as a potential conflict of interest.
